# Qualifications and Competency Needs among Professionals in Outpatient Care for Young People with Co-occurring Problems

**DOI:** 10.1177/14550725251384006

**Published:** 2025-10-27

**Authors:** Sabina Kapetanovic, Sabina Vlasman, Karin Boson, Peter Wennberg, Mats Anderberg, Mikael Dahlberg

**Affiliations:** 1 Department of Behavioral Sciences, 42749 University West, Trollhättan, Sweden; 2 Department of Psychology, 3570 Gothenburg University, Gothenburg, Sweden; 3 Department of psychology, 3207 University of Inland Norway, Lillehammer, Norway; 4 Department of Public Health Sciences, 7675 Stockholm University, Stockholm, Sweden; 5 Department of Social Work, 4180 Linnaeus University, Växjö, Sweden; 6 Department of Pedagogy, 4180 Linnaeus University, Växjö, Sweden

**Keywords:** competency needs, co-occurring problems, Maria clinics, treatment, substance use

## Abstract

The study explores the qualifications and competency needs of professionals working at Swedish Maria clinics, specialized in treating youth with co-occurring substance use and mental health problems. A web-based survey was conducted among 87 professionals working at the clinics to assess their qualifications, competence in managing co-occurring problems and perceived needs for further training. Most professionals had high educational qualifications, with backgrounds in social work, nursing and psychology. Although health care and social services professionals had generally similar competencies, there were some notable differences in their professionalism and competency needs. Healthcare professionals were more likely to report competence in managing severe psychiatric conditions. In contrast, social services professionals more often reported using a broader range of treatment methods. Both groups identified a need for further education in managing severe psychiatric conditions, such as trauma, psychosis and eating disorders. Social services professionals more frequently emphasized the need for family-oriented approaches, while healthcare professionals more often identified a need for more in-depth knowledge related to substance use. While professionals at the clinics generally possess strong qualifications, there are differences in their confidence and expertise relating to managing complex mental health conditions. Tailored training initiatives that address specific needs based on professionals’ educational backgrounds and organizational affiliations could enhance inter-professional collaboration and improve treatment outcomes for youth with co-occurring substance use and mental health problems.

Substance use problems among young people are a growing concern and are often compounded by co-occurring mental health issues, including anxiety and neuropsychiatric disabilities. In Sweden, approximately 34–54% of young people in outpatient clinics for substance use problems report undiagnosed mental health problems such as sleeping issues, depressive symptoms and trauma, while approximately 20% receive medication for psychiatric disorders and ongoing psychiatric care (Richert et al., 2020). The presence of both diagnosed and undiagnosed mental health problems during treatment for substance use disorders significantly increases the risk of dropout and unfavorable outcomes (Hulvershorn et al., 2015), which highlights the need for a greater focus on co-occurring mental health problems. In response to the complex and often co-occurring substance use and mental health problems among adolescents, the Swedish regions and municipalities co-manage Maria outpatient clinics, which are specialized in interdisciplinary treatment centers and designed to provide integrated care. Run by regional healthcare and municipal social services, the clinics enable coordinated medical, psychological and social interventions, offering services such as psychosocial assessments, drug testing, manualized treatments, family interventions and psychiatric consultations. However, the extent to which professionals working in these clinics are equipped to manage co-occurring substance use and mental health problems remains unclear. A sense of competence can strengthen clinical confidence, enhance practitioner engagement, and support the delivery of effective interventions (Sacks et al., 2013). Therefore, to better understand the readiness of professionals to address the co-occurrence of substance use and mental health problems in an integrated treatment setting, the present study explores the qualifications and competency needs of professionals working in outpatient clinics for youth substance use treatment.

Internationally, and in Sweden, the prevalence of mental health issues such as anxiety and neuropsychiatric disabilities is notably high among youth and young adults with substance use problems (Bender et al., 2006; Morisano et al., 2014; Spencer et al., 2021). These overlapping problems are often referred to as a comorbidity or co-occurring problems. These individuals typically have more severe alcohol and drug use, with an earlier onset and higher frequency of use (Bertrand et al., 2013; Deas, 2006; Godley et al., 2014). The co-occurring problems are also considered to generate more severe social problems such as criminality, family conflicts and school issues. Although some studies suggest that there are few differences between young people with and without co-occurring mental health problems relating to treatment engagement and outcomes (Battjes et al., 2003; Godley et al., 2014; Tanner-Smith et al., 2013), other studies indicate that, relative to individuals with only substance use problems, patients with co-occurring problems are more likely to drop out from the treatment, relapse and have poorer treatment outcomes (Hawkins, 2009; Hulvershorn et al., 2015; Jacobsson et al., 2011). Given the severe implications and complexities of co-occurring mental health and substance use problems, enhancing our understanding and treatment approaches for this vulnerable population is imperative.

Overall, three different treatment approaches with interventions for co-occurring mental health problems in substance use treatment are described in the literature (Hogue et al., 2018; Morisano et al., 2014): 1) sequential treatment, where one condition is addressed before the other. This approach, however, lacks evidence for positive outcomes; 2) parallel treatment, where the individual receives support for both conditions simultaneously from separate units with no collaboration between mental health services and substance use treatment (such an approach is considered risky as no provider has overarching responsibility for both problems); and 3) integrated treatment where both conditions are addressed simultaneously within the same unit. This approach has been shown to be the most suitable model for young people with co-occurring problems and has strong scientific support (Hogue et al., 2018; Morisano et al., 2014; Torchalla et al., 2012).

Although there are some indications for effective approaches for treating co-occurring substance use and mental health problems, co-occurring mental health problems are rarely detected within the healthcare system (Hawkins, 2009; Priester et al., 2016). One possible reason is the fragmented nature of the healthcare system, typically divided among different authorities (Hawkins, 2009). This, in turn, can lead to delays in necessary treatment interventions, interventions occurring in the wrong order, or patients being unable to receive appropriate support: “Clients, in turn, often are caught in the middle of this split, experiencing what has been termed ‘ping-pong therapy’ as they are bounced back and forth between mental health services and addiction settings” (Minkoff, 2000). Additionally, it is not uncommon that each authority demands one condition being fully treated before the other can be addressed (Priester et al., 2016), which essentially delays, or has negative impact on the efficacy of the overall treatment outcomes. Notably, the responsibility distribution between social services and health care regarding interventions and costs are uncertain and conflicted (Sacks et al., 2013). The responsibility for coordinating contact with the services as well as coordinating the interventions is usually placed on the patients themselves or their families, while engagement level among decision-makers within the healthcare system to establish this type of service for the target group is rather low (Sacks et al., 2013). As a result, an excessive specialization within substance use treatment, both internationally and in Sweden, has occurred at the expense of a more holistic approach where both conditions are simultaneously addressed (Hawkins, 2009; Petersén & Berman, 2023).

Notwithstanding, treatment of co-occurring mental health problems in adolescents and young adults is crucial to optimize the substance use treatment outcomes and for achieving maximum benefit in the care of mental health conditions (Bender et al., 2006; Morisano et al., 2014). Generally, there is a lack of integrated and specialized services that treat co-occurring problems (Priester et al., 2016; Sacks et al., 2013). Professionals with relevant competence in substance use problems are often not present within psychiatric units and, conversely, while many professionals working in specialized substance use care have general knowledge of mental health issues, there is often limited access to staff with advanced or specialized training in the treatment of complex psychiatric conditions. There is also a major lack of primary and further education addressing the knowledge and the treatment of co-occurring problems (Hawkins, 2009). This type of integrated approach and methods are used to a very limited extent even in Sweden, despite strong recommendations in the Swedish national guidelines (National Board of Health and Welfare, 2019; see also Petersén & Berman, 2023). Nevertheless, one positive example of suitable, integrated care units that are designed to meet the multidimensional needs of young people with substance use problems are the so called “Maria clinics” (National Board of Health and Welfare, 2022; SALAR, 2018; SOU, 2020; 2021a; 2023).

The specialized Maria clinics are based on close cooperation between social services and health care. In recent years, clinics have been established in several cities, either under regional (i.e., health care) or municipal (i.e., social services) organizations. There are approximately 50 Maria clinics in Sweden (SOU, 2021b, 2023). Most clinics provide psychosocial and medical assessments related to substance use, drug testing, individual or family therapy, and manualized treatment programs. Depending on the young person's problems and the chosen treatment model, practitioners from different professions, including social workers, nurses, psychologists and doctors, may also work together on shared cases. On average, patients remain in treatment for approximately 4–6 months. The size of the local unit varies; in larger cities, the staffing is 10–20 people, while, in a smaller city, there may be three to six staff (Anderberg & Dahlberg, 2018). Consequently, the professionals working in these settings come from two different organizational contexts that may differ in terms of training, mandate and treatment methods. The professionals belonging to the regional organization are located within the healthcare system, while those under the municipal organization fall under the jurisdiction of social services. Regardless of organizational affiliation, they often encounter young people with co-occurring mental health problems and substance use (Richert et al., 2020). However, it is unclear what knowledge and skills they have when it comes to treating co-occurring conditions.

The service delivery, knowledge and competence of professionals working with young people who have co-occurring problems can be understood through the lens of organizational theories. For example, applying the theories of knowledge-based work (Evetts, 2013), it remains to be explored to what extent professionals working at Maria outpatient clinics align with *occupational* or *organizational* professionalism. Occupational professionalism is defined by autonomy, specialized expertise, ethical standards and collegial control, where professionals themselves regulate their practice and uphold standards, as seen with professions like nurses and psychologists. In contrast, organizational professionalism involves a higher degree of external oversight from politicians or authorities, characterized by standardized procedures, administrative supervision and adherence to organizational goals and guidelines, which is common in more bureaucratic settings like social work and teaching. In the present study, the concepts of occupational and organizational professionalism are used as an interpretive lens to better understand the reported competencies and organizational differences between professionals employed by health care and social services in Maria outpatient clinics.

The aim of this study is to describe and analyze the qualifications and the competence development needs among professionals working at Maria clinics. In addition, the study also explores potential differences between professionals employed in regional health care versus those in municipal social services, reflecting the dual organizational structure of Maria outpatient clinics. The following research questions are guiding our study:

1. What qualifications, both general and specific, do professionals in Maria clinics possess?
2. What treatment methods are being used, and how do professionals perceive their competence in dealing with co-occurrent conditions?
3. What are the competency needs of professionals at Maria clinics?
4. What are the similarities and differences between professionals from the sectors of social services and health care in Maria outpatient clinics?

## Methods

The present study is a part of the research project Treatment Research on Adolescents at the Maria clinics (TRAM). The primary purpose of the project is to study the developmental trajectories of adolescents within the Swedish Maria substance use outpatient clinics. The particular focus of the project is on alcohol and drug use, mental health, social situation, and what role specific risk and protective factors play in term of outcomes for various groups post outpatient treatment (Anderberg et al., 2019; Anderberg et al., 2021). Moreover, the project also highlights the qualifications and competencey needs concerning co-occurring mental health and substance use problems amongst the professionals at the Maria clinics in Sweden. The study has been ethically approved (Ref. no. 2015/160-31).

### Procedure

Data were collected between October and November 2023. The unit managers in 16 large, medium-sized and small cities received a letter containing information about the study, along with a request to schedule a telephone call. These calls involved providing additional details about the study, inviting participation and offering the managers the opportunity to ask questions about the study. They subsequently distributed a survey link to professionals at their respective clinics, accompanied by information about the study and instructions that emphasized the anonymity and voluntary nature of participation. No personal information, including the names of clinics or cities, was collected. All the invited clinics agreed to participate.

### Participants

The survey was distributed to a total of 107 professionals, out of which 82 provided data, forming the analytic sample for the study. The gender distribution is 82% females and 18% males, with the mean age of 45 (range 27–66 years). The participants had an average of five years of experience working at the Maria clinic and an average of 15 years of experience in their profession. Out of all participants, 68% were employed by social services and 32% by healthcare organizations. While there is no national data specifying a standard occupational division in Maria clinics, this distribution may reflect common organizational arrangements in which clinics are primarily administered by social services in many municipalities.

### Materials

The data was collected through a web-based survey consisting of 46 questions, including both fixed-response and open-ended items. The survey, which took around 10 min to complete, covered several thematic areas. For the purpose of this study, five of these areas were selected for further analyses. These areas include: 1) background data: e.g., gender, age, and education; 2) perceived competence in working with young people with different psychiatric conditions; 3) psychosocial treatment methods used in clinical settings; 4) interventions and support aimed at family and relatives; and 5) perceived competence development needs. The survey was purpose-developed for this study to capture relevant information about professionals’ competence and practices within Maria outpatient clinics. The content of the questionnaire was informed by national guidelines for youth substance use treatment, prior research on co-occurring substance use and mental health problems, and consultation with clinical experts working in Maria outpatient settings.

### Data Analysis

Descriptive statistical analysis was conducted to summarize the demographic characteristics and key variables of the sample. Chi-squared tests were employed to examine associations between categorical variables, allowing for the identification of statistically significant differences within the data. In addition to the quantitative analysis, a content analysis was performed on the open-ended responses. Following the recommendations from scholars (Hsieh & Shannon, 2005; Schreier, 2012), the content analysis followed a systematic procedure beginning with familiarization, where the open-ended responses were reviewed multiple times to identify initial themes. Preliminary codes were then developed based on recurring words, phrases and ideas. The entire dataset was systematically coded, with each response potentially receiving multiple codes. These initial codes were grouped into broader categories that captured higher-level concepts. The frequency of each code and category was counted, providing insight into the prevalence of specific ideas.

## Results

Table 1 presents the general and specific qualifications of the professionals in the study.

**Table 1.** Educational Background by Professionals from Social Services and Healthcare (N = 82).

	Total (%)	Social Services (%)	Healthcare (%)
University degree	94	93	96
Social work education	52	66	23
Psychology education	7	2	19
Bachelor's degree in behavioral/social education	17	25	0
Nursing education	17	0	54
No university degree	6	7	4
Further education	74	79	65

*Note*. Further education included training in treatment methods and specialized university courses relevant to their field.

As shown in Table 1, the professionals working in the Maria clinics are highly educated, with the majority (94%) holding a university degree, primarily in social work, nursing, other behavioral/social science (i.e., behavioral science, social pedagogy or sociology) and psychology. Professionals employed in social services preferably worked as therapists or social workers, while those employed within health care were nurses or psychologists. Additionally, three-quarters of the participants (74%) have pursued further education in treatment-specific methods such as cognitive behavioral therapy (CBT) and motivational interviewing (MI). Some of them had also undertaken further education in the form of university courses relevant to their profession, such as criminology and addiction.

At the Maria clinics, professionals meet young people with substance use problems and various types of psychiatric conditions. We therefore asked about their ability to handle these types of problems (Table 2).

**Table 2.** The Percentage of Professionals Who Feel Competent in Working with Specific Mental Health Conditions (N = 82).

	Total (%)	Social Services (%)	Healthcare (%)	*p*-Value
ADHD	85	86	85	0.896
Anxiety disorder	83	77	96	**0**.**030**
Depression	76	70	88	0.065
Conduct disorder	71	70	73	0.750
Suicidal ideations	71	64	85	0.060
ASD	66	71	54	0.118
Self-harm	65	57	81	**0**.**037**
Other NPD	50	57	35	0.058
OCD	46	43	54	0.353
Trauma/stress	43	38	54	0.164
Personality disorder	43	34	62	**0**.**019**
Bipolar disorder	37	30	50	0.086
Eating disorder	30	30	31	0.970
Psychosis	26	20	38	0.069

*Note.* The psychiatric conditions are ordered based on the level of perceived competence. ADHD = attention deficit hyperactivity disorder; ASD = autism spectrum disorder; NPD = neuropsychiatric disorders; OCD–obsessive–compulsive disorder. Significant *p*-values are presented in bold.

As noted in Table 2, most professionals reported having competence in managing young people with psychiatric conditions such as attention deficit hyperactivity disorder (ADHD), anxiety and depression. Fewer professionals, however, reported competence in managing more complex conditions such as bipolar disorder, eating disorders and psychosis. A larger proportion of healthcare professionals reported competence in handling psychiatric conditions compared to their counterparts in social services, with significant differences observed for anxiety, self-harm and personality disorders. Somewhat unexpectedly, a greater proportion of social services professionals reported competence in working with neuropsychiatric conditions compared to those in health care.

We also analyzed which different psychosocial methods the professionals used in their work (Table 3).

**Table 3.** Psychosocial treatment methods used by professionals (N = 82).

	Total (%)	Social Services (%)	Health Care (%)	*p*-Value
Motivational interviewing (MI)	90	95	81	**0**.**049**
Relapse prevention (RP)	79	84	69	0.127
Hashish withdrawal program (HAP)/cannabis program for				
young people (CPU)	62	73	38	**0**.**003**
Cognitive behavioral therapy (CBT)	52	52	54	0.862
Functional family therapy (FFT)	32	41	12	**0**.**007**
Adolescent community reinforcement approach (ACRA)	30	34	23	0.321
Family therapy (not specified)	27	32	15	0.111
Motivational enhancement therapy (MET)	26	32	12	**0**.**047**
Community reinforcement approach and family training				
(CRAFT)	10	14	0	**0**.**042**
Dialectical behavior therapy (DBT)	7	5	12	0.317
Aggression replacement training (ART)	5	7	0	0.162
Other treatment methodsª	8	7	12	0.507

*Note*. The methods are ordered based on the level of frequency of use reported by the clinicians; ª Examples of other treatment methods are Mindfulness and programs for criminality. Significant *p*-values are presented in bold.

The four most prominently reported treatment methods were MI, relapse prevention (RP), hashish withdrawal program/cannabis program for young people (HAP/CPU) and CBT. There were also a substantial proportion of professionals who reported different types of family-oriented treatment methods, more than two-thirds in total. Manual-based methods such as functional family therapy (FFT) and community reinforcement approach and family training (CRAFT), as well as more unspecified family therapeutic methods, were also mentioned. Professionals employed in social services reported using MI, HAP, FFT, motivational enhancement therapy (MET) and CRAFT, significantly more often than their counterparts in health care.

The second part of the results addresses the professionals’ competency needs with respect to both psychiatric conditions and general educational efforts for the current target group (Tables 4 and 5).

**Table 4.** The Prevalence of Perceived Competency Needs Regarding Psychiatric Conditions Among Professionals (N = 82).

	Total (%)	Social Services (%)	Health Care (%)	*p*-Value
Eating disorder	85	88	81	0.422
Psychosis	84	88	77	0.222
Trauma/stress	84	86	81	0.568
Personality disorder	80	82	77	0.579
OCD	79	88	62	**0**.**007**
Bipolar disorder	77	82	65	0.094
Self-harm	77	82	65	0.094
Other NPD	76	73	81	0.458
Conduct disorder	71	75	62	0.213
Suicidal ideations	70	70	69	0.970
ASD	65	70	54	0.164
Depression	63	71	46	**0**.**027**
Anxiety disorder	57	64	42	0.061
ADHD	56	62	42	0.086

*Note.* The methods are ordered based on the frequency level of reported needs for competence development by the professionals. ADHD = attention deficit hyperactivity disorder; ASD = autism spectrum disorder; NPD = neuropsychiatric disorders; OCD–obsessive–compulsive disorder. Significant *p*-values are presented in bold.

**Table 5.** Overarching Categories for Suggested Competence-Enhancing Training Initiatives (N = 82).

	Total (%)	Social Services (%)	Health Care (%)	*p*-Value
Family-oriented approaches	29	38	12	**0**.**016**
Psychosocial interventions	29	32	23	0.401
Mental health problems	27	27	27	0.990
Co-occurring problems	12	12	12	0.901
Substance use problems	11	4	27	**< **.**001**

*Note.* The categories in the table are mutually exclusive. Significant *p*-values are presented in bold.

The psychiatric conditions for which the need for knowledge and competence development was most commonly reported for eating disorders, psychosis and trauma/stress-related disorders. In contrast, the need for additional knowledge was less prevalent for conditions such as depression, anxiety disorders and ADHD. A greater proportion of professionals in social services reported a need for competence development compared to those in health care, with significant differences observed in relation to depression and obsessive–compulsive disorder (OCD).

To further investigate the professionals’ competence development needs, an open question was asked: *If you could determine the content of a competence-enhancing training initiative for yourself and your colleagues – what would it include and focus on?* To analyze the qualitative data collected from this open-ended question, a content analysis method was employed. The content analysis identified five overarching and mutually exclusive categories, as detailed in Table 5 below.

The first category includes 29% of the professionals who expressed a need for competence development in *family-oriented approaches*. These professionals emphasized the importance of engaging with families to achieve sustainable change in young people's lives. For instance, one professional from social services highlighted the need for training on “meeting families with psychiatric difficulties” and stressed the significance of “working with them, for example, through family counseling”. Notably, professionals from social services were significantly more likely to request this type of competence development compared to their counterparts in health care.

Education on *psychosocial interventions* and strategies for working with young people was sought by 29% of the professionals. One professional from social services expressed a need for “clarity on how to approach working with young people on behavioral change”, while another professional from the healthcare sector required “emotion regulation strategies/early interventions for personality disorders as well as psychoeducational interventions for ADHD” to improve therapeutic engagement with young people. Professionals from social services were more likely to express a need for psychosocial interventions compared to those in health care.

About 27% of the professionals expressed a desire for competence development in treatment and understanding of *mental health problems*. For example, there was a need for competence development in terms of responding to psychiatric diagnoses in general, as well as specifically understanding neuropsychiatric disorders and intellectual disability. One professional from social services highlighted a lack of competence as well as resources for guiding young people and families following receiving a diagnosis from the psychiatric care, stating: “Child and adolescent psychiatric care provides diagnoses but lacks the knowledge and resources to support the young person effectively following the diagnosis”. Another professional from social services expressed the need for information on “which treatment interventions have strong evidence, such as skills in dialectical behavior therapy (DBT) for personality disorders, obsessive–compulsive disorder (OCD), eating disorders and psychoses”. In this category, no significant differences were observed based on organizational affiliation.

Competence development in terms of *co-occurring problems* was emphasized by 12% of the professionals, who specifically expressed a need for deeper knowledge of the nature of co-occurring conditions and effective strategies for their management in clinical practice. One professional from social services underscored the importance of increased knowledge about collaboration between the two organizations noting that “… this is where the challenges arise, more so than the competence of individual therapists”. Other professional from the healthcare sector requested more comprehensive education on “how we, as an integrated organization, can develop our methods and capabilities to address co-occurring problems in young people alongside substance use” and called for educational programs on university level. In this category, the desire for competence development was consistent across different organizational groups.

More need for competence of *substance use problems* was stated by 11% of the professionals. In this context, there was a pronounced emphasis on understanding addictive conditions and their consequences. One professional from social services noted: “As a newcomer, I need to acquire a comprehensive understanding of various substances and addiction illnesses to effectively perform my tasks”, while another professional from the healthcare sector asked for more “competence of the biological aspects of substance use and addiction”. Additionally, yet another professional from the healthcare sector requested “advanced education in addiction and co-occurrence of mental health problems, ideally tailored for nurses and generating university credits”. Healthcare employees demonstrated a significantly higher demand for competence development related to substance use problems compared to their counterparts in social services.

## Discussion

Substance use problems that co-occur with mental health problems, such as anxiety and neuropsychiatric disabilities, are a growing global concern. In Sweden, this challenge is particularly pronounced because these conditions are often addressed separately by two different organizations: social services and healthcare (SOU, 2023). To meet the complex needs of young people facing both substance use and mental health problems, specialized interprofessional outpatient clinics, known as Maria clinics, have been established. The aim of this study was to explore the qualifications and competency needs of professionals working within both health care and social services in these integrated treatment settings.

Exploring the basic qualifications among professionals in the Maria clinics, we found that the level of their education background is notably high, with almost all respondents holding a university degree. Among the social service professionals, the majority have an educational background in social work, while those working within the healthcare system are primarily trained as nurses or psychologists. Additionally, many have pursued further education in areas directly relevant to their work, such as studies in criminology and addiction. The majority had also received specialized training in treatment-specific methods. Given that adolescents enrolled in Maria outpatient clinics often present with complex, multidimensional issues, including childhood trauma and criminal behavior (Anderberg et al., 2019, 2022), the advanced and specialized competencies of these professionals may be well-positioned to effectively address the diverse needs of their young clients. These educational backgrounds align with the basic competencies recommended for implementing knowledge-based approaches and treatment methods for the target population (SOU, 2023). This level of inter-professionalism is essential for effectively addressing co-occurring problems of substance use and mental health problems (Morisano et al., 2014).

The healthcare staff is dominated by nurses who mainly work at the Maria clinics as professionals in integrated teams in accordance with recommendations from previous research (Hogue et al., 2018; National Board of Health and Welfare, 2019). In some cases, they are not always a part of an interprofessional team, which means they are mostly engaged in their traditional healthcare tasks, such as health dialogues, checkups, urine sampling and medication.

Professionals working at Maria clinics report competence in managing young people with co-occurring substance use and conditions such as ADHD, anxiety and depression, but fewer report confidence in handling bipolar disorder, eating disorders and psychosis. The management of these severe psychiatric conditions is inherently more demanding for both the youth and the professional, requiring a deeper level of experience and specialized knowledge in treatment strategies (Adams, 2008; McKee, 2017). Notwithstanding, a greater proportion of healthcare employees report competence in managing both severe and less severe psychiatric conditions compared to their counterparts in social services. This discrepancy may reflect differences in professional training and institutional orientation, consistent with the framework of knowledge-based work and the distinction between occupational and organizational professionalism (Evetts, 2013). Social workers, whose practice is rooted in relationship-building and a holistic understanding of individuals’ social contexts (Johnson et al., 2023), often operate within systems characterized by administrative oversight and standardized procedures. This organizational logic may limit opportunities for specialized clinical training and reduce professional discretion. In contrast, healthcare professionals typically work in environments that support greater autonomy, access to specialized resources and structured clinical pathways (Lim et al., 2022). These conditions align more closely with occupational professionalism, which emphasizes expert knowledge, professional autonomy and peer regulation. Thus, the higher proportion of healthcare staff reporting competence may reflect the greater professional discretion and specialized training inherent in the healthcare system.

A large majority of professionals in the study (range 52–90%) report that they use manual-based treatment methods in their clinical work. Several of these treatment methods are recommended in the Swedish national guidelines, such as MI, MET, CBT and an adolescent community reinforcement approach (ACRA). Additionally, and according to the recommendations by the national guidelines (National Board of Health and Welfare, 2019), more than two-thirds use family-oriented methods, such as FFT and CRAFT, where parents or siblings of the patient are involved in the treatment. The HAP and CPU programs, mentioned by several respondents, are two treatment methods that have their theoretical basis in CBT. These programs are developed in Sweden but lack sufficient evidence among young people with substance use problems (National Board of Health and Welfare, 2019). Despite these weak recommendations, the methods have become very widespread in Sweden (Patriksson, 2014). One possible explanation is that the development towards an evidence-based practice in Sweden generally has a significantly weaker anchoring in social services than in health care (SOU, 2023). The use of such methods as DBT and aggression replacement training (ART), particularly when working with adolescents with multidimensional problems and needs, may enhance the overall effectiveness of treatment by addressing a broader range of behavioral and emotional issues (Hogue & Dauber, 2013). This approach could potentially lead to more comprehensive care and better outcomes for the young people treated at these clinics.

The professionals from social services report employing a broader range of treatment methods compared to their counterparts in health care. This finding is likely reflective of the broader scope of challenges that social services professionals encounter. Social services often engage with vulnerable families facing multifaceted issues, such as poverty, trauma and social instability, which demand a more diverse toolkit of intervention strategies (Van Eck et al., 2024). This diversity in approach could be essential for addressing the underlying social determinants of health that contribute to substance use and other related problems. By utilizing a variety of methods, the professionals at the Maria clinics can offer more tailored and responsive care, potentially leading to more holistic and sustainable outcomes for their patients.

Regarding the need for knowledge and competency development related to psychiatric conditions, professionals from social services most frequently identified eating disorders, psychosis and trauma- or stress-related problems. For conditions such as depression, anxiety and ADHD, the need for additional knowledge is perceived as less important. There are also differences, with social service professionals generally reporting a greater need for competence development on psychiatric conditions compared to healthcare workers. Although the proportion of young people with co-occurrent conditions decrease significantly as a result from a 3-year follow-up study in Maria clinics, mental health problems persist for one-third of the participants (Dahlberg et al., 2022). This trend is more pronounced among young women than young men. Hence, it is not surprising that the majority of the professionals in the present study identify a need for knowledge development related to some of the most severe psychiatric conditions among their clients.

Finally, professionals were asked an open-ended question about their needs for competence-enhancing education. Five key areas emerged from the analysis: family-oriented approaches, psychosocial interventions, mental health problems, co-occurring problems and substance use problems. Professionals from social services more frequently emphasized the need for training in family-oriented approaches, likely reflecting their focus on addressing the complex, multidimensional challenges faced by the young people they support (Almqvist & Lassinantti, 2018). The expressed desire for further training in psychiatric conditions and manual-based treatment methods among social service professionals may reflect an aspiration toward greater occupational professionalism, characterized by increased autonomy, specialized expertise, and professional discretion (Evetts, 2013). In contrast, healthcare professionals more often highlighted the need to deepen their knowledge of substance use, addiction, and its neurobiological and behavioral consequences, reflecting a domain-specific orientation consistent with their clinical role and training context.

While the study provides valuable insights into the qualifications and competency needs among professionals working at Maria outpatient clinics, certain limitations must be acknowledged. First, while the sample includes professionals from 16 clinics of varying sizes and organizational structures, not all Maria clinics in Sweden are represented. This limits the generalizability of the findings, especially in relation to smaller or more rural clinics that may have different resources or organizational challenges. Second, the sample size, although sufficient for the descriptive and comparative analyses conducted, may not capture the full diversity of experiences and practices across all clinics. Third, the study relied on a purpose-developed questionnaire rather than validated instruments to measure perceived competence, which may affect the reliability and comparability of the results. In addition, most competence-related items used binary (yes/no) response options, which may oversimplify nuanced perceptions of skill and confidence. Finally, the cross-sectional design limits any conclusions about causality between organizational affiliation and perceived competence or development needs.

### Implications

Overall, the findings underscore the importance of integrating diverse therapeutic approaches and interpersonal strategies when designing comprehensive training initiatives to enhance knowledge among professionals in Maria clinics (Riggs, 2003; Sacks et al., 2013). It is clear that the professionals indicate notable competency needs in family-oriented treatment methods, which is to be expected considering the organization's formal mandate and its specific target group. Addressing these needs requires the development of targeted educational initiatives, which, for specialized clinics working with young people's substance use treatment, should ideally consist of multiple packages or modules. These should build on existing qualifications and competencies at the same time as remaining aligned with the professionals’ educational backgrounds. Considering the differences in both educational training and organizational goals between social services and healthcare staff, such initiatives need to be tailored to their specific professional contexts. Ultimately, interventions that integrate competency needs with educational and professional backgrounds have the potential to strengthen interprofessional collaboration and empower practitioners to provide more informed and effective care for young clients with substance use and co-occurring mental health problems.

### Conclusions

The present study provides valuable insights into the competencies and needs of professionals working with co-occurring substance use and mental health problems. Across educational backgrounds, professionals demonstrate high general and formal competence in treating this target group. However, their confidence in managing severe psychiatric conditions and in applying various treatment methods differs depending on professional background. Social services professionals report somewhat lower confidence in treating severe psychiatric conditions but tend to employ a broader range of treatment methods, reflecting the diverse challenges they face in their work. Healthcare professionals, by contrast, report greater competence in managing psychiatric conditions, likely due to their clinical training and access to specialized resources. Despite these differences, both groups identify similar areas for further development, including family-oriented approaches, treatment of specific psychiatric conditions, and enhanced knowledge about substance use. These findings suggest that differences in competencies and training needs may be shaped by the professionals’ distinct organizational affiliations, educational backgrounds, and professional perspectives. Training and interventions should therefore be tailored to these specific contexts to strengthen interprofessional collaboration and enable more comprehensive care for young people with complex, multidimensional needs, including co-occurring substance use and mental health problems.
